# «Cognitus & Moi»: A Computer-Based Cognitive Remediation Program for Children with Intellectual Disability

**DOI:** 10.3389/fpsyt.2016.00010

**Published:** 2016-02-03

**Authors:** Caroline Demily, Caroline Rigard, Elodie Peyroux, Gabrielle Chesnoy-Servanin, Aurore Morel, Nicolas Franck

**Affiliations:** ^1^GénoPsy, Center for the Diagnosis and Management of Genetic Psychiatric Disorders, CH Le Vinatier, Bron, France; ^2^UMR 5229, EDR-Psy, Center of Cognitive Neuroscience, CNRS, University Lyon 1, Lyon, France; ^3^Centre référent lyonnais en réhabilitation psychosociale et en remédiation cognitive (CL3R), CH Le Vinatier, Bron, France; ^4^Unité de Psychoéducation Polaire, Centre Hospitalier le Vinatier, Bron, France

**Keywords:** attention, visuospatial functions, social cognition, cognitive remediation, intellectual disability, behavior, hyperactivity

## Abstract

Attentional, visuospatial, and social cognition deficits have a negative impact on children’s adaptative and social competences and, as a result, on their ability to achieve a normal functioning and behavior. Until now and despite the frequency of those deficits, there is a lack of children’s specific cognitive remediation tools specifically dedicated to attentional and visuospatial areas. The «Cognitus & Moi» program involves a variety of exercises in a paper and/or pencil (*n* = 30) or a computerized format (*n* = 29) and a strategy coaching approach. Each module of «Cognitus & Moi» targets a single impaired cognitive area, within the limits of cognitive domains’ overlapping. The little cartoon character named Cognitus, who illustrates the program, is supposed to be very friendly and kind toward children. Cognitus will accompany them throughout the program for an effective and positive reinforcement. The main goal of «Cognitus & Moi» is to adjust to children’s difficulties in daily life. Moreover, since the cognitive remediation benefit is complex to apply in daily life, the program is based on a metacognitive strategy. After a complete neuropsychological assessment and a psychoeducational session (with the child and the parents), 16 1-h-sessions of cognitive remediation with the therapist are proposed. Each session is composed of three parts: (1) computerized tasks focusing on specific attentional or visuospatial components (20 min). The attentional module targets hearing, visual, and divided attention. A double attention task is also proposed. The visuospatial module targets eye tracking and gaze direction, spatial orientation, visuospatial memory and construction, and mental imagery; (2) pen and paper tasks focusing on the same processes (20 min) and a facial emotion recognition task; (3) a proposal of a home-based task (during 20 min). Weekly, specific attentional and visuospatial home tasks are proposed to the child and analyzed with the parents and the therapist. Indeed, home exercises are useful to promote the transfer of strategies to daily life and their subsequent automation. The heterogeneity of cognitive deficits in intellectual deficiency necessitates an individualized cognitive remediation therapy. In this regard, «Cognitus & Moi» seems to be a promising tool.

## Why Should We Consider Cognitive Remediation for People with Intellectual Disability?

### Definition and Functional Outcome

In most classifications, such as the DSM-5 (1), intellectual disability is defined as a deficit of cognitive abilities such as reasoning, problem solving, planning, abstract thinking, judging, academic learning, and learning by experience. However, other cognitive dysfunctions are associated with intellectual disability. Actually, attentional (2), memory (3), visuospatial (4), and executive (5–7) alterations are also found.

All these can be associated with an impairment of adaptive functioning. This impairment prevents people from being independent and socially responsible and appears during the development stages. Research on the functional and concrete impact of these cognitive deficits is currently being structured and developed. As an example, Berg (8) and Bull and Scerif (9) have shown that the mathematical difficulties encountered by children with intellectual disabilities are due to their low working memory capacities and to an executive dysfunction. In effect, working memory and short-term memory play a major role in a number of daily activities (10) because they help maintain and control information in memory (11). Thus, we may conclude that cognitive deficits are closely related to the functional outcome.

The care of people with intellectual disability is a public health issue, 1–3% of the population being concerned (12). Speech therapy and psychomotor care are well-developed for children with intellectual disability (13). These techniques mainly help these people develop their communication and motor skills. Since cognitive functions other than language (14, 15) and psychomotor abilities are impaired in children, as well as adults, it seems interesting to consider the development of specific cognitive remediation programs.

### Definition of Cognitive Remediation

Cognitive remediation is a behavioral training aiming at minimizing the daily impact of cognitive deficits by optimizing and improving cognitive functioning (16). In this regard, the notion of functional adaptation should be highlighted. In this connection, cognitive remediation has been defined as psychological treatment aimed at “increasing the general cognitive efficiency to improve global adaptation, independence, and well-being.”

The development of new strategies and tasks can be used to improve cognitive functioning. In the field of intellectual disability, two theories explain the effectiveness of cognitive remediation.

– The first one is based on a developmental approach (17). People with intellectual disability follow the same development as people without but at a slower pace. In this case, cognitive remediation is used to acquire the main skills such as categorization, spatial relations, and the numbering and ordering concepts.
– The second theory is based on a modular conception of cognition (18). People with intellectual disability present various cognitive profiles: preserved skills as well as deficits can be observed. Differences depend on the origin of the deficit. For example, diverse cognitive profiles can be observed according to the genetic conditions (Williams syndrome, Down syndrome, or X-Fragile syndrome…) (5, 19). Those data have mainly been shown in the field of memory (20). In this case, cognitive remediation can help develop strategies to improve impaired cognitive processes. The «Cognitus & Moi» program is based on this conception.

## Cognitive Remediation of Children Affected by Intellectual Disabilities: The Different Programs

Concerning intellectual disability, cognitive remediation programs must be based on some general principles. They should be clearly defined before beginning the treatment. The therapist ought to set objectives according to the child’s practical needs. Improvement of cognitive functions will enable the child to reach his/her goals.

### Memory Programs

Cognitive remediation focused on short-term memory has been well studied. Hulme and Mackenzie (21) worked on articulatory recapitulation strategy using a cumulative repetition method. It consisted of ten 10-min sessions. This program was carried out with teenagers (13–18 years old) affected by intellectual disabilities. The authors showed a significant improvement of the memory span. Then, Comblain (22) enhanced this program by proposing 30-min individual weekly training sessions (growing difficulty) with subjects (*n* = 12) affected by Down syndrome. This study showed a significant improvement of the average memory span. Moreover, long-term effects were also observed 18 months later. With this strategic approach, Bussy et al. (23) demonstrated an extension of the verbal span and a growing passive vocabulary. Finally, improvements were observed in the verbal short-term memory of 33 children suffering from fetal alcohol syndrome (24).

In the field of meta-memory, Kendall et al. (25) have shown that children with intellectual disability are able to learn an interrogative strategy encouraging them to find links between different stimuli. This group showed higher scores than the control one as far as recalling items were concerned.

### Metacognitive Program

Découvrez vos capacités, rEalisez vos possibilités, pLanifiez votre démarche, soyez créatiFs (DELF) is a metacognitive program, which aims at discovering the subject’s skills and highlighting the person’s abilities. This program is used in groups and aims at teaching metacognitive strategies (anticipation, planning, and control) and more specific strategies helping, for example: to better use working memory so as not to overload memory. DELF proved to be effective with teenagers suffering from intellectual disability when associated with a regular teaching program (26). Within this experimental group, inductive thinking abilities were improved.

### Virtual Reality

Few studies focus on the use of virtual reality for the treatment of people with intellectual disabilities (27). Rose et al. (28) pointed out the relevance of an active exploration in a virtual environment with the use of a joystick rather than a passive exploration thanks to a mere observation. Handling a joystick enabled the subjects to better memorize the environment visuospatial data. Thus, development of virtual reality programs would enable patients to improve their visuospatial abilities.

### Attentional Program

Galbiati et al. (29) proposed treatment for children and teenagers aged 6–18 years, presenting traumatic brain injury combined with a mild intellectual disability and attentional difficulties. The program used targeted attentional abilities by using metacognitive strategies. Treatment lasted for 6 months, including four 45-min individual weekly sessions led by a therapist. During the sessions, 30 min were dedicated to computerized exercises and 15 min to paper/pencil exercises. The participants’ attention resources and adaptive skills improved on a daily basis.

## Visuospatial Skills and Social Cognition in Intellectual Disability

Visuospatial ability refers to the capacity to identify visual and spatial relationships among objects. How subjects imagine objects, perceive global shapes, and how they locate small components or understand the similarities and differences among objects are major cognitive functions.

Visuospatial and visuoperceptual skills play a key role in everyday life. Visual information and complex visual stimuli are analyzed with a complete unawareness of the visuoperceptual process or the complexities of the stimuli involved. This process becomes conscious in a context of learning. Repetition and familiarity enable a more spontaneous approach and turn the conscious and effortful process into an automatic one.

If this ability is impaired, many types of deficits can occur, ranking from a failure to process the basic elements of a visual stimulus (i.e., colors, lines, orientation) to more complex and integrative features such as object identification, faces, or familiar scenes. These deficits can include social cognition defects, especially in the area of facial emotion recognition.

Social cognition is a psychological construction referring to the understanding of others’ thoughts and including several components such as empathy, attribution bias, theory of mind, and emotion processing. Impairments in this field may largely underlie social dysfunctions and reduce adaptive skills. Moreover, social cognitive disabilities contribute more or less directly to behavioral disturbances and psychiatric symptoms (e.g., depression, anxiety) (30). Yet, depressive and anxious symptoms are often found in children with intellectual disabilities (31, 32). In this regard, a link is clearly established between children’s language and behavioral impairments when it comes to intellectual disability (33). Behavioral and psychiatric symptoms are also correlated to intellectual disability in adults, as shown by Deb et al. (34).

Children with externalizing behavioral problems provide aggressive responses to hypothetical vignettes more spontaneously than children with intellectual disability without any behavioral problems (35). Cognitive dysfunctions explain the hardship children encounter in the treatment of social information (36). Indeed, social cognition dysfunction appears like a core symptom (37). The theory of mind and facial emotion recognition seem to be central for social adaptation to the environment (38).

As far as social cognition is concerned, the facial emotion recognition is well documented. Children with intellectual disability fail to recognize and match emotional facial expressions from a series of photographs depicting various facial expressions (39). This deficit is correlated with an abnormal behavior (40) and underlain by visuospatial and attentional deficits (41).

In conclusion, the improvement of attentional and visuospatial deficits thanks to a specific cognitive remediation program could have a positive impact on the children’s social cognition and behavior. This approach would complete the methods already available.

## The «Cognitus & Moi» Program

Attention, visuospatial, and social cognition deficits have a negative impact on children’s adaptive and social competences and, as a result, on their ability to achieve normal functioning and behavior. Until now and despite the frequency of those deficits, there is a lack of children’s specific cognitive remediation tools specifically dedicated to attentional and visuospatial areas.

The «Cognitus & Moi» program targets attentional and visuospatial functions (Table 1). Cognitive goals are embedded in two different modules (attention and visuospatial) and the level of these modules is chosen according to the child’s key difficulties. Each exercice of «Cognitus & Moi» targets a single-impaired cognitive area.

**Table 1:** **«Cognitus & Moi»: attentional and visuospatial modules**.

Attention	Visuospatial
Hearing attention	Eye tracking/gaze direction
Visual attention	Spatial orientation
Divided attention	Visuospatial memory
Double attentional tasks	Mental imagery
	Visuospatial construction

«Cognitus & Moi» was developed in France through the collaboration between the GénoPsy center (*Center for the Detection and Management of Psychiatric Genetic Disorders*) in Lyon, the EDR-Psy research team (CNRS & Lyon 1 University, headed by Pr. Nicolas Franck) and the SBT Company (headed by Pr. F. Tarpin-Bernard). The Génopsy team examines patients who suffer from psychiatric genetic disorders. Thus, neurocognition, social cognition, as well as metacognition are routinely evaluated. The SBT Company and the EDR-Psy team have already developed various cognitive remediation programs (RECOS, GAIA, RC2S) (42, 43).

All the developers implicated in «Cognitus & Moi» are trained therapists for cognitive remediation. The exercises of the attentional and visuospatial modules were selected in the SBT software database. First, the selected exercises were proposed to children with normal cognitive functioning in order to appreciate the technical feasibility. Second, each exercise was adapted (levels one to nine) in order to be proposed to children with intellectual disability. The third step was the development of a facial emotion recognition task. Each Cognitus’ facial expression was based on the Ekman pictures (*Appendix 4* in Supplementary Material) with three levels of intensity (44).

«Cognitus & Moi» The «Cognitus & Moi» program involves a variety of exercises in a paper and/or pencil (*n* = 30) or a computerized format (*n* = 29) and a strategy coaching approach. Therapists make use of techniques known to benefit the rehabilitation of cognitive syndromes.

This new cognitive remediation program was elaborated with two main goals in mind. The first one aimed at adjusting to the children’s difficulties in daily life. In order to achieve this purpose, we developed an individualized and flexible program meant to improve both specific attentional and visuospatial impairments and to reach each child’s concrete objectives. Since the cognitive remediation benefit is complex to apply in daily life, a program based on collaboration with parents was elaborated (Table 2).

**Table 2:** **Keys of the program**.

«COGNITUS & MOI»: main principles
• Intensive and targeted cognitive training
• Therapist and child relationships: interactive process
• Learning modalities:
– Active processing in addition to practice
– Verbal mediation techniques,
– Training of processes implied in attentional and visuospatial functions
– Selecting relevant information
• Concrete goals
• Content: 2 independent fields (attentional and visuospatial processes) totalizing 29 exercises
• Modalities: paper and pencil + computerized training
• A collaborative therapy: development of child’s own strategies
• Target: selective attentional and visuospatial impairments
• Adaptability: nine levels of difficulty for each exercise
• Exercises adapted to each child’s capacities including a positive reinforcement
• Exercises with parents at home (supervised by the therapist)
• Psychoeducation: for both the child and his/her parents at the beginning of the therapy

«Cognitus & Moi» is designed for 5–13-year-old children with or without intellectual deficiency. The little cartoon character named Cognitus, who illustrates the program, is supposed to be very friendly and kind toward children. Cognitus will accompany them throughout the program for an effective and positive reinforcement.

### Cognitive Assessment

A complete and detailed neuropsychological assessment prior to the cognitive remediation treatment seems necessary (45). This evaluation will also determine the severity of the impairments and their impact on everyday life. Currently, consensus lacks concerning attentional and visuospatial evaluations, so they reflect the heterogeneity of the performances.

This assessment must help establish the global degree of intellectual disability by using standardized tools such as the Wechsler scales. Then it is important to assess the functional level of the person by using more specific tools such as the Vineland Adaptative Behavior Scale, second Edition [VABS-II (46)], the EFI [Functional Intervention Scale (47)] or the AAPEP [Psycho-educative profile for adolescents and adults (48)]. In intellectual disability, the IQ is most often evaluated without a thorough evaluation of the different cognitive domains. However, a detailed cognitive assessment is necessary to establish a detailed neuropsychological profile by identifying the cognitive impairments and also the preserved abilities of the person.

The following assessment is recommended (before the «Cognitus & Moi» program): (Table 3).

**Table 3:** **Neuropsychological assessment**.

Domains	Tests	Targeted functions
Cognitive performances	WPPSI III (56) or WISC IV (57)	Intellectual abilities
Language	Peabody Picture Vocabulary Test-R (58)	Passive vocabulary
Praxis	Imitating hand positions – NEPSY II [(59, 60); French adaptation: ECPA]	Gestural praxis
Visuospatial processing	Visuomotor precision – NEPSY II	Oculomotor coordination
	Sky search – TEA-Ch (61)	Visual search/spatial selective attention
	Arrows – NEPSY II	Judgment of line orientation
	Block construction – NEPSY II	Visuospatial construction
	Route finding – NEPSY II	Visuospatial interactions
Memory	Word lists – CMS [(62); French adaptation: centre de psychologie appliquée]	Verbal memory
	Dot locations – CMS	Spatial memory
Attention	Alertness – kiTAP	Vigilance/visual attention
	Auditory attention and response set part 1 – NEPSY II	Selective auditory attention
	Divided attention – kiTAP/TAP	Divided attention
Executive functions	Go/NoGo – kiTAP (63)/TAP [(64); French adaptation: Leclercq M.]	Motor inhibition
	Auditory attention and response set part 2 – NEPSY II	Cognitive inhibition and cognitive flexibility
	Inhibition – NEPSY II	Cognitive inhibition
	Labyrinths – WISC III	Visuospatial planning
Social cognition	Theory of mind – NEPSY II	Theory of Mind
	Affect recognition – NEPSY II	Facial affect recognition

*CMS, Children’s Memory Scale; NEPSY II, *Developmental NEuroPSYchological Assessment (second edition);* TAP/kiTAP, test battery of attention performance; WISC IV, Wechsler Intelligence Scale for Children (fourth edition); WPPSI III, Wechsler Preschool and Primary Scale of Intelligence (third edition)*.

### Preliminary Session: Psychoeducation

In the first part of this session, the therapist, the parents and the child go through the previously administered assessment together. By explaining the child’s cognitive assessment to the parents helps them better understand his/her profile, and which of his/her cognitive components are impaired and which are preserved. That is the reason why this session lays emphasis upon the specific impairment in the child’s daily life.

The last session of the preparation phase provides psychoeducation via two specific documents. A specific comic strip has been elaborated for children explaining the main goals of attentional and visuospatial functions (*Appendix 1* in Supplementary Material). Moreover, a didactic document «Parent handbook» is proposed to the parents in order to detail the attentional and visuospatial functions and the «Cognitus & Moi» program (*Appendix 2* in Supplementary Material).

The aim of the psychoeducational session is to allow the child and his/her parents to understand the specific terms used in the field of neurocognition, their implication in daily life, to finally increase motivation.

### Cognitive Remediation Sessions with the Therapist

Then, 16 1-h-sessions of cognitive remediation with the therapist are proposed, each session is composed of three parts:

(1) pen and paper tasks focusing on specific attentional or visuospatial component (20 min),
(2) computerized tasks focusing on the same process (20 min),
(3) a proposal of a home-based task (during 20 min).

Each of the 16 sessions deals with a specific attentional and visuospatial stimulation. The computer-based tasks are ranked in increasing order of difficulty, with nine levels of complexity. This allows the therapist to adapt difficulty of exercises to the child’s abilities.

### Design of the Computer-Based Modules (*n* = 29) (Table 4)

#### Design of the Pen-and-Paper Based Modules (Table 5)

Similarly, pen-and-paper exercises will help the child to develop compensatory strategies when it comes to visuospatial or attentional tasks. Adapting the rhythm of the treatment to the child’s abilities helps prevent him/her being faced to failures. The rhythm is progressive, as must be the difficulty of each exercise.

**Table 4:** **Computer-based modules**.

For example: visual attention	
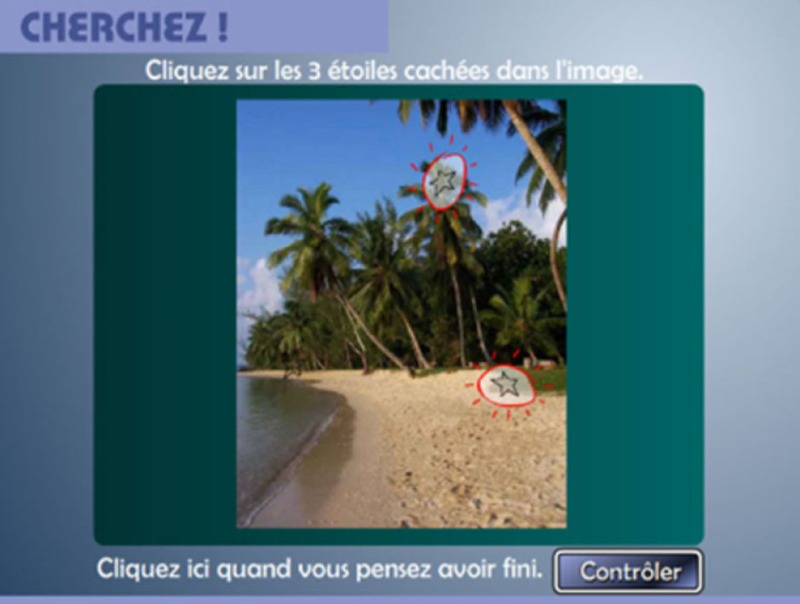	***“Looking for”:* selective attention, visual exploration and discrimination**
	Several identical symbols are hided in a photography. The child has to observe the image and to locate the symbols. The complexity of the task increases according to the determined level (one to nine).
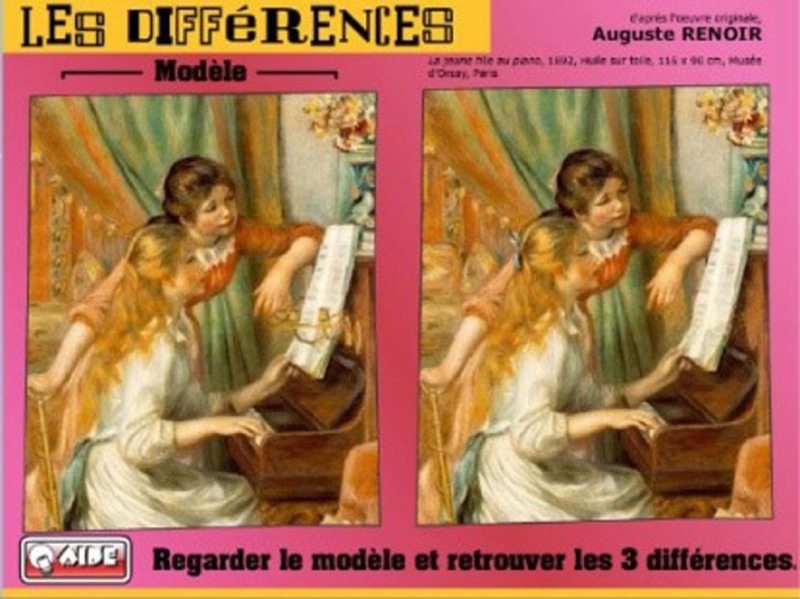	***“The differences”*: selective attention, visual exploration and discrimination**
	A child, with the help of a therapist, will determine the difference between the two sides of the presentation. He/She will have to find which image appears on the right but not on the left. The child should use a specific strategy to answer properly.

**Table 5:** **Pen-and-paper based modules**.

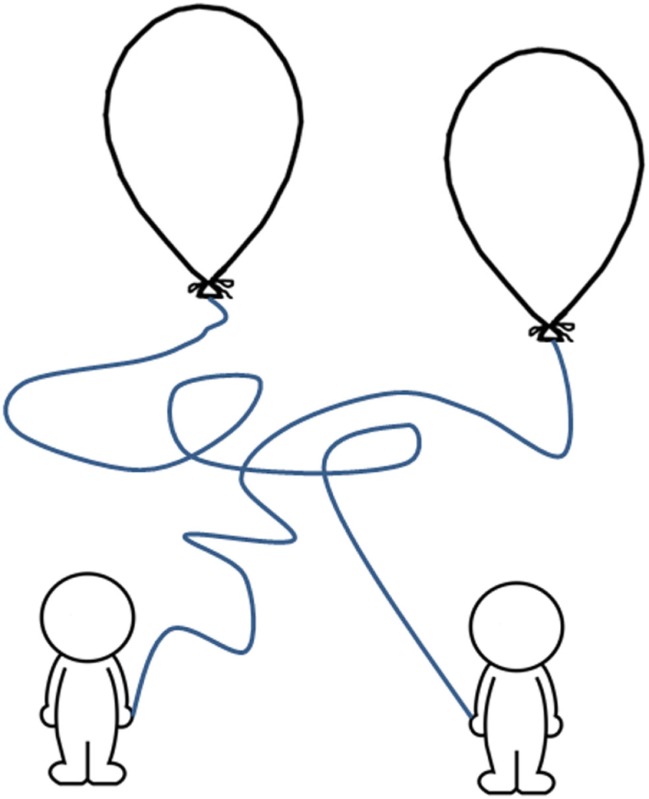
The child has to follow and color the link between the characters and the balloons

Finally, the child will have to recognize Cognitus’ facial emotion (*Appendix 4* in Supplementary Material) with the help of the therapist. This exercise was developed in order to establish a concrete link between visuospatial functions and facial emotion recognition. The drawings are designed in order to encourage the person to focus on the eyes. People with intellectual disabilities often study the mouth area to recognize emotions. The strategy developed in this tool will help children determine an emotion by using relevant information. By highlighting the eye area, the strategy can be automated by the child. He/She has to choose the correct emotional label by verbalizing the name of the emotion. Six universal emotions are represented (without neutral condition): happiness, fear, anger, disgust, surprise, and disgust.

### Therapist’s Strategies in «Cognitus & Moi»

Mediation by a therapist is one of the key factors to the success of psychological treatment. Individual care helps maintain the collaboration over time between the child and the therapist. Verbalization plays an active part in the training of a new strategy. The therapist helps the child find sense when a problematic situation arises. The development of the new skills will be transferred to daily life. The therapist should encourage the child to actively take part in the treatment by choosing his/her own program of exercises and goals.

The structuration of the sessions is required in order to help the child deal with the notion of delay and waiting. This can be done by presenting the program of each session at the beginning and by estimating the time to be spent on each task. After each task, the therapist will remark on the time spent on the exercise to improve the perception of chronological indicators.

This technique is detailed in different steps in «Cognitus & Moi», in order to automatize and generalize the new strategies.

– First, the therapist describes out loud the different steps composing the used strategy.
– Then, it is up to the child to describe these steps out loud.
– Finally, the child must be able to internalize these steps without the help of the therapist.

Children with an intellectual disability will find it easier to transfer memory strategies to daily life when it has been explicitly verbalized during the cognitive remediation sessions (49).

### Home Task with the Parents

The cognitive remediation session with the therapist finishes with a proposal of a home task. Exercises take into account both the level of performance of the child, the collaboration of the parents, and are related to the concrete objectives defined at the beginning of the therapy.

The outstanding idea of the program is to establish a weekly link between cognitive remediation and daily life. Moreover, we will help the child understand his/her environment using colors and time landmarks. Tasks are adapted to the child’s needs and take into account his/her daily reality. For example, at the beginning of the therapy, it could be suggested that the child should shop at the local supermarket and find a given item in a department.

Later, specific attentional and visuospatial home tasks can be proposed to the child and analyzed with the parents and the therapist. Finally, tasks such as the planning of a daily route can be suggested. Home exercises are useful to promote the transfer of strategies to daily life and their subsequent automation. This is consistent with learning theories. Indeed when cognitive remediation is reinforced in real-world settings, the learning process is facilitated and then generalization and transfer are promoted (50). The child and the therapist will go through the home task together at the beginning of the next remediation session. In order to promote motivation during the home task, the use of a personalized exercise book allows a concrete weekly evaluation of the degree of achievement of the task. The child can self-evaluate his/her performances with a smiley, in the same way, his/her parents can also evaluate their child by answering four concrete questions:

1. Did your child complete the activity?
2. How did your child find the activity?
3. How did your child behave during the activity?
4. Was the goal of the activity clear to you?

## Metacognitive Aspects and Benefits

People with intellectual disabilities have difficulties in tasks requiring a voluntary effort and a conscious analysis of the cognitive demands of the task. This difficulty prevents the subjects from spontaneously engaging themselves in a task when this one requires a strategic active treatment.

Metacognition corresponds to the knowledge of one’s own functioning. The metacognitive processes are procedural aptitudes that have an impact on the cognitive aims of the person and on the tasks carried out. In this regard, positive reinforcement represents a key to success.

In our program, Cognitus the little character, kindly supports the child (*Appendix 3* in Supplementary Material). Moreover, the parents’ cooperation plays a key role. That is the reason why, «Cognitus & Moi» is clearly distinguished from the daily homework to avoid conflicts.

Only few patients with intellectual disabilities manage to find work or live alone; however, families hope that close relatives will be able to be more independent (27). This is why the ultimate aim of a cognitive remediation program is to improve the subject’s quality of life. This has to be done by acquiring new strategies that will help the patient achieve the exercises and then generalize the strategies and benefits. The idea is to apply a learned strategy to a more global context, distant from the initial one. People with intellectual disabilities are described as cognitively passive (51). This is characterized by a lack of strategy transfer (52). It is particularly complicated to obtain transfer; each strategy taught has a different adaptive value depending on the environment and on the needs of the person (53). It is important to first acquire the skills before trying to contextualize them in more complex environments. The therapist will help systematically to establish the link between the skills acquired during the sessions and situations of daily life by using, for example, home tasks. These tasks should first be defined with the patient during the sessions and a feed-back will then be given during the following session.

In general, children with intellectual disabilities have a sense of failure and the feeling of being incompetent. Cognitive remediation helps the child escape this negative spiral. Positive reinforcement and mediation by a therapist in the «Cognitus & Moi» program have an impact on the child’s confidence in his/her abilities and thus on self-esteem. In this regard, the child is the winner of an award after the cognitive remediation training (*Appendix 3* in Supplementary Material).

Self-determination is the possibility for the person to choose his/her own activities and behaviors (54). The therapist plays an important part in the improvement of self-determination. By reinforcing the child’s feeling of competence (positive feedback) and by choosing adapted exercises (nor too easy, nor too difficult) the therapist improves self-confidence (55).

## Validation Study

The cognitive deficits presented by patients with intellectual disability are very diverse. Yet, most studies on the effectiveness of cognitive remediation treatment are randomized controlled trials, especially in the area of mental health and education. Randomized controlled trials have been used to evaluate a number of educational interventions but remain less frequent in the cognitive remediation area.

We are currently conducting (preliminary steps) a randomized controlled trial in order to establish the validity of the «Cognitus & Moi» program. All the ethical approval (French legislation) is in process (CPP, CCTIRS, CNIL, and Clinical trials.gov registration). Our first objective is to evaluate the impact of the program on behavioral disorders. A thorough assessment is proposed including a complete evaluation of the components of neurocognition, social cognition, and of social functioning and behavior. These measures and the complete assessment are repeated at the end of the intervention to highlight the impact of the «Cognitus and Me» program, and 6 months later to investigate the possible long-lasting effects of the benefits. Currently, the first children are following the therapy with the «Cognitus and Me» program in a context of usual care. The exercises appear very close to daily life and very pleasant. Another point is the parents’ wishes to be involved in their child’s therapy. In this regard, the program meets their expectations.

However, our program presents several limits. First, only the attentional and visuospatial deficits as well as emotion recognition are targeted. Other cognitive dysfunctions are associated with intellectual disability, especially memory, and executive functions. Indeed, the development of these specific modules will be the next step of the «Cognitus & Moi» program. Second, the involvement of other components of social behavior (social skills training and management of emotions) plays a key role in the adaptative skills. In that regard, cognitive behavioral therapy should be a useful treatment in addition to the «Cognitus & Moi» program. Third, to our knowledge, the long-term effects of cognitive remediation in intellectual deficiency have not been investigated in previous studies.

## Conclusion

This article opens the path for new thoughts about the development of specific cognitive remediation program in intellectual disability. A new modular conception of cognitive remediation in intellectual deficiency should include neurocognition, social cognition and metacognition training programs. Cognitive remediation tools, based on visuospatial and attentional functions, constitute promising tools to improve social cognition in patients with intellectual disability and behavioral disorder. The aim of this work is to enable these people to have access to cognitive remediation treatments that are specifically adapted to their abilities and needs.

Intellectual disability is a public health issue and it is essential to consider all the possible solutions to help improve these people’s everyday life. Cognitive remediation is thus one of the most promising tools that can be used. However, it must be used with a very strict methodology in order to respect the person’s limits and preserved skills. Last but not least, cognitive remediation must be included in a global care of the person and cannot be substituted to other psychological treatments. This treatment depends on the participation of different actors (e.g., family, therapist, educator, etc.). The interaction between the different medical and social actors will help establish a complementarity among the different treatments carried out (medical treatment, psychomotor, speech therapy…). To conclude, the «Cognitus & Moi» program, by using transfer strategies to daily life, is close to the real world. This allows the child to practice skills in specific personal contexts, which is a major challenge to improve mental resources.

## Author Contributions

CD, EP, CR, AM, and GC-S were implicated in the concept and the design of «Cognitus & Moi». CD, CR, and EP wrote the paper. GC-S and NF were involved in the review of the literature. All authors read and approved the final manuscript.

## Conflict of Interest Statement

The authors declare that the research was conducted in the absence of any commercial or financial relationships that could be construed as a potential conflict of interest.
