# Supplementary material for: «Cognitus & Moi»: A Computer-Based Cognitive Remediation Program for Children with Intellectual Disability
Source: Front Psychiatry. 2016 Feb 3;7:10. doi: 10.3389/fpsyt.2016.00010 (PMC4737901; doi:10.3389/fpsyt.2016.00010)
Supplement: Supplementary file 1 [file image_1.pdf]

Cognitus and me

My home workbook

I am

Child's photo
2" x 2"

____

My cognitive remediation partners are

Cognitus and ___________

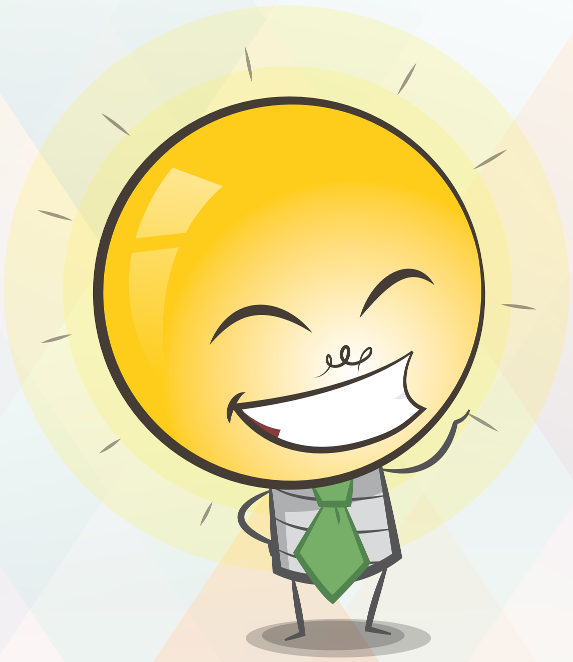

Therapist's photo
2" x 2"

What is attention?

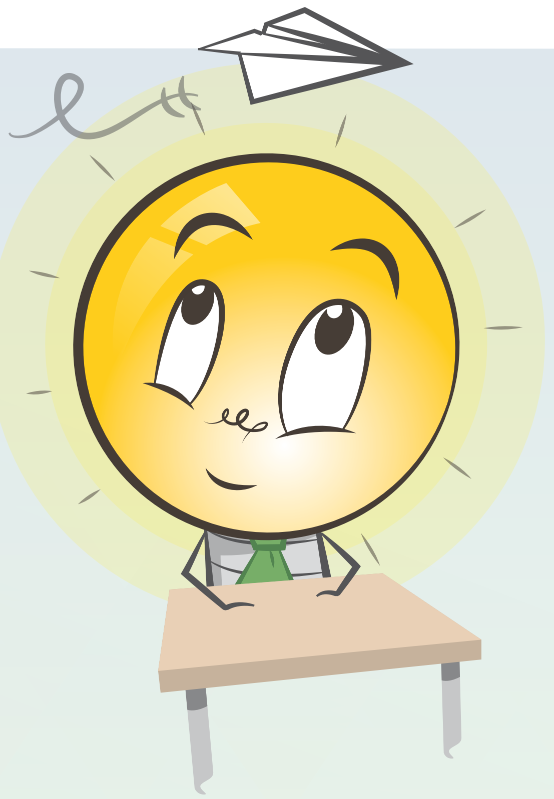

Why do we need it to understand our location in space?

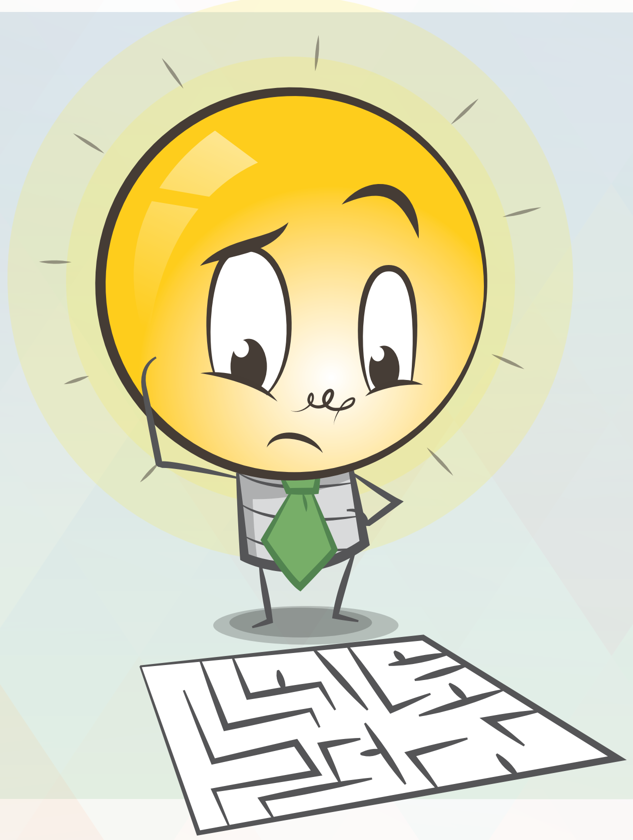

You have completed a neuropsychological assessment.

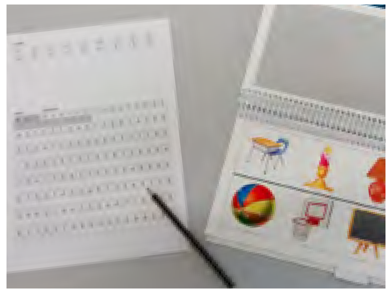
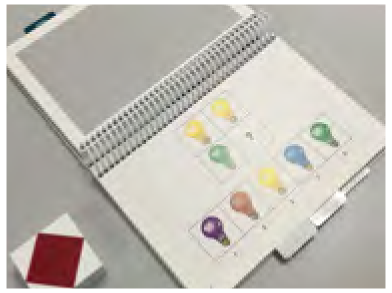
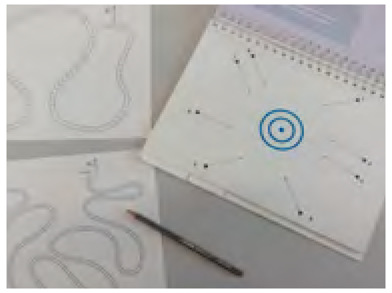

The assessment showed that you were very good at:

But that other areas were more difficult for you:

With cognitive remediation, Cognitus will help you find strategies to overcome your difficulties.

Cognitive remediation

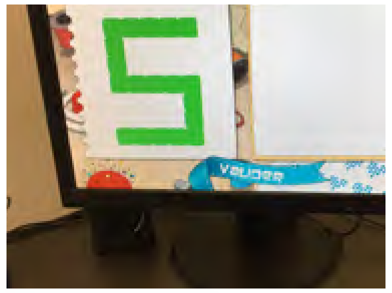
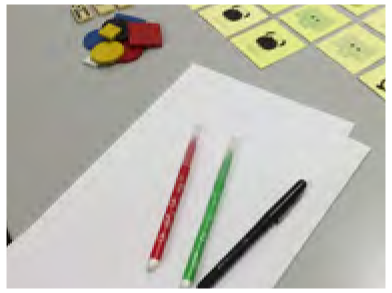
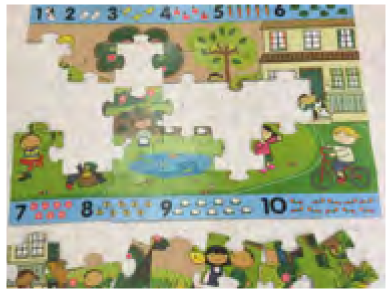

You'll be coming once a week to do computer activities and other activities with pencil and paper.

It will be a fun time during the week when you'll have a chance to develop your strengths. Cognitus is here to support and encourage you.

You will also have activities to do at home with your parents. Through these activities, you'll be able to take the things you do with Cognitus and try them out in your daily life.

Session calendar	
Day	Time

Date : ___ / ___ / ___

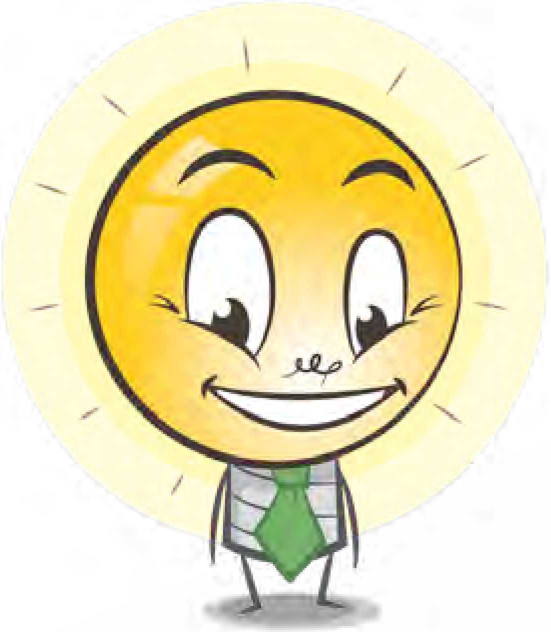

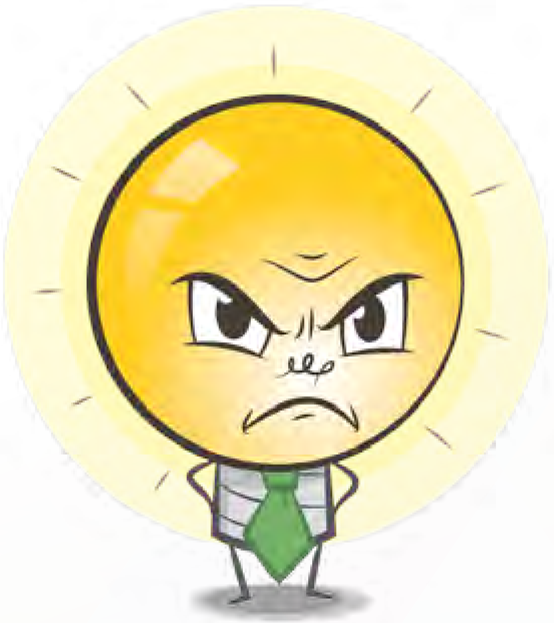

What am I doing this week?

How did my home activity go?

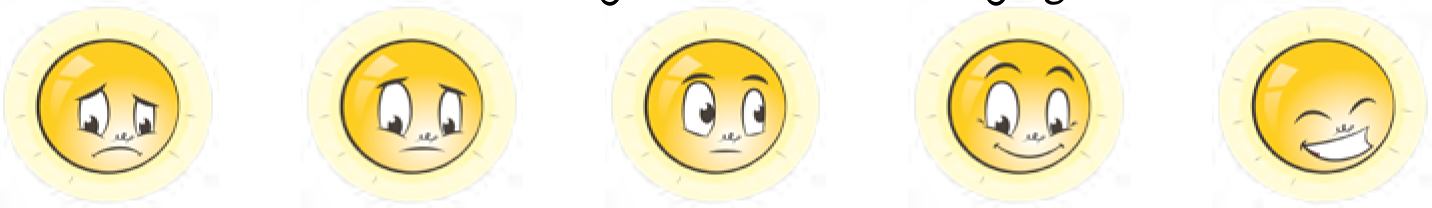

Section to be completed by parents.

Did your child complete the activity?

☐ Yes ☐ No ☐ Partially

How did your child find the activity?

☐ Very easy ☐ Easy ☐ Appropriate ☐ Difficult ☐ Very difficult

Was the goal of the activity clear to you?

☐ Yes ☐ No ☐ Partially

How did your child behave during the activity?

☐ Very restless ☐ Restless ☐ Indifferent ☐ Focused ☐ Very focused

Comments:

Date : __ / __ / __

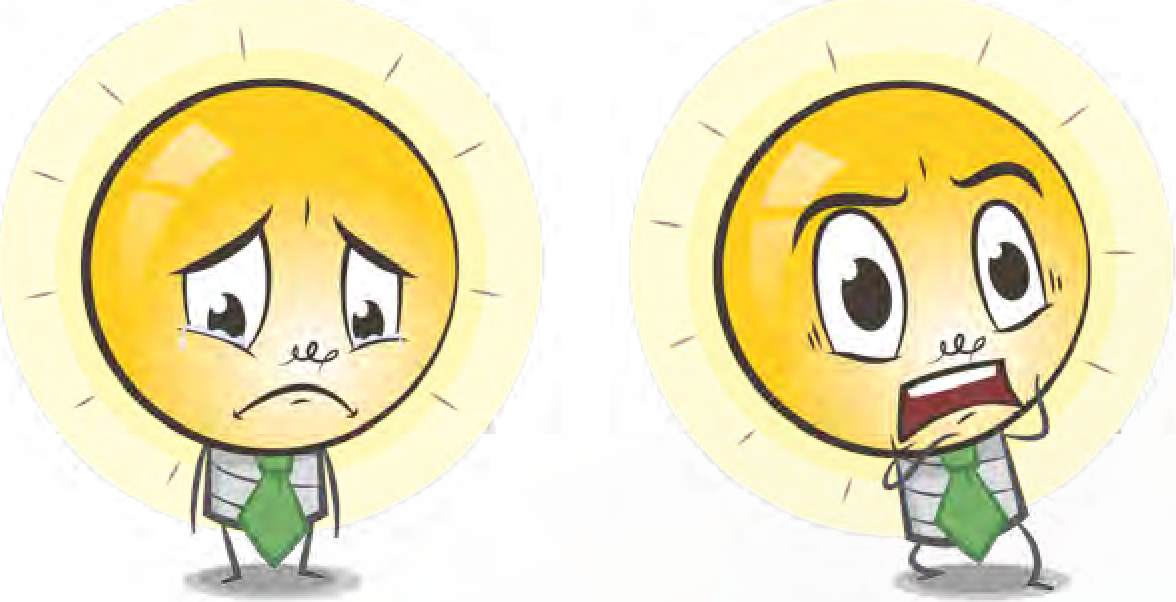

What am I doing this week?

How did my home activity go?

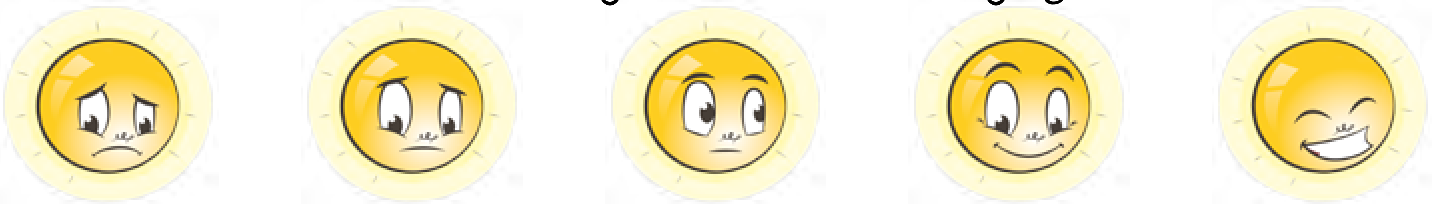

Section to be completed by parents.

Did your child complete the activity?

☐ Yes ☐ No ☐ Partially

How did your child find the activity?

☐ Very easy ☐ Easy ☐ Appropriate ☐ Difficult ☐ Very difficult

Was the goal of the activity clear to you?

☐ Yes ☐ No ☐ Partially

How did your child behave during the activity?

☐ Very restless ☐ Restless ☐ Indifferent ☐ Focused ☐ Very focused

Comments:

Date : ___ / ___ / ___

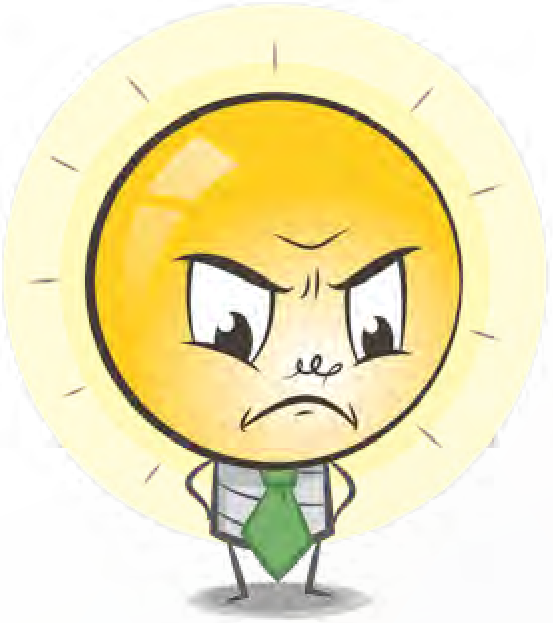

What am I doing this week?

How did my home activity go?

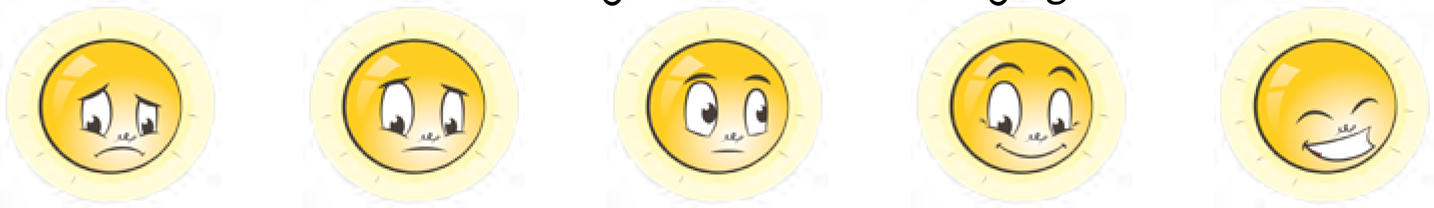

Section to be completed by parents.

Did your child complete the activity?

☐ Yes ☐ No ☐ Partially

How did your child find the activity?

☐ Very easy ☐ Easy ☐ Appropriate ☐ Difficult ☐ Very difficult

Was the goal of the activity clear to you?

☐ Yes ☐ No ☐ Partially

How did your child behave during the activity?

☐ Very restless ☐ Restless ☐ Indifferent ☐ Focused ☐ Very focused

Comments:

Date : __ / __ / __

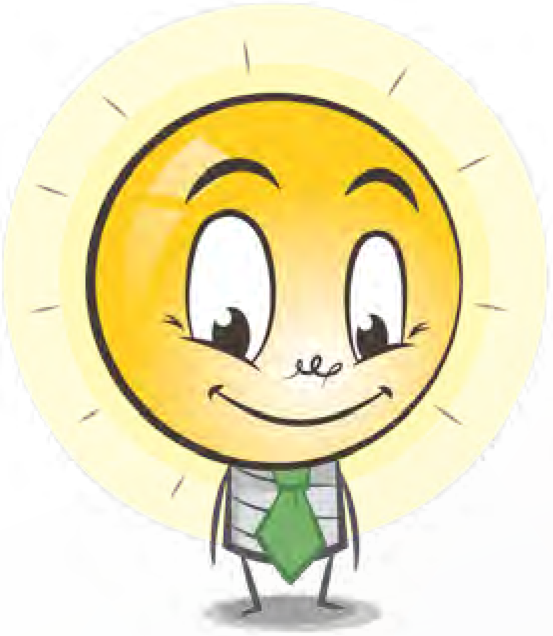

What am I doing this week?

How did my home activity go?

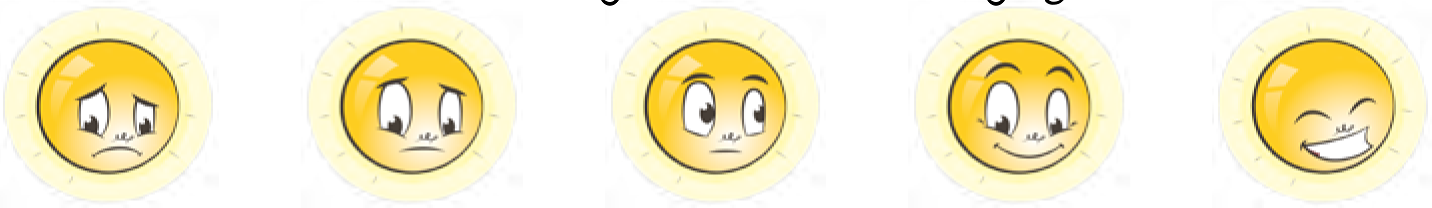

Section to be completed by parents.

Did your child complete the activity?

☐ Yes ☐ No ☐ Partially

How did your child find the activity?

☐ Very easy ☐ Easy ☐ Appropriate ☐ Difficult ☐ Very difficult

Was the goal of the activity clear to you?

☐ Yes ☐ No ☐ Partially

How did your child behave during the activity?

☐ Very restless ☐ Restless ☐ Indifferent ☐ Focused ☐ Very focused

Comments:

Date : ___ / ___ / ___

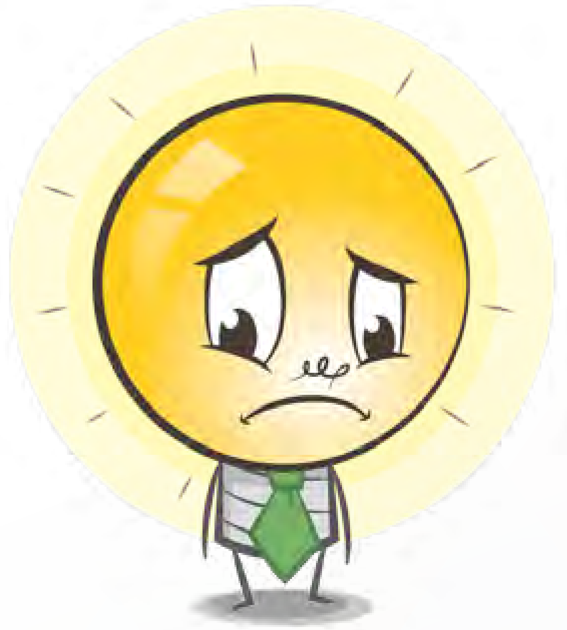

What am I doing this week?

How did my home activity go?

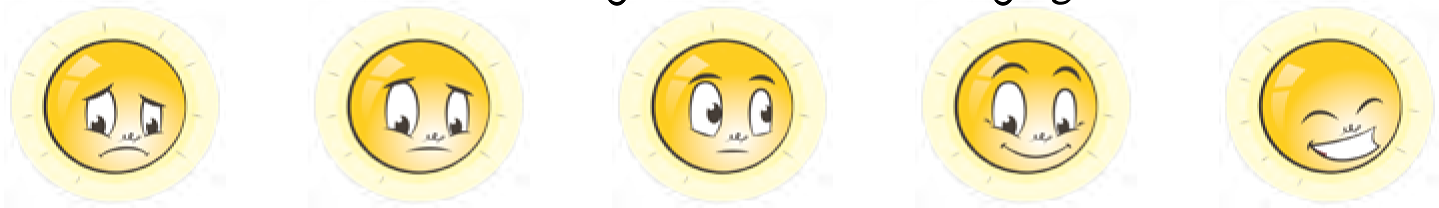

Section to be completed by parents.

Did your child complete the activity?

☐ Yes ☐ No ☐ Partially

How did your child find the activity?

☐ Very easy ☐ Easy ☐ Appropriate ☐ Difficult ☐ Very difficult

Was the goal of the activity clear to you?

☐ Yes ☐ No ☐ Partially

How did your child behave during the activity?

☐ Very restless ☐ Restless ☐ Indifferent ☐ Focused ☐ Very focused

Comments:

Date : __ / __ / __

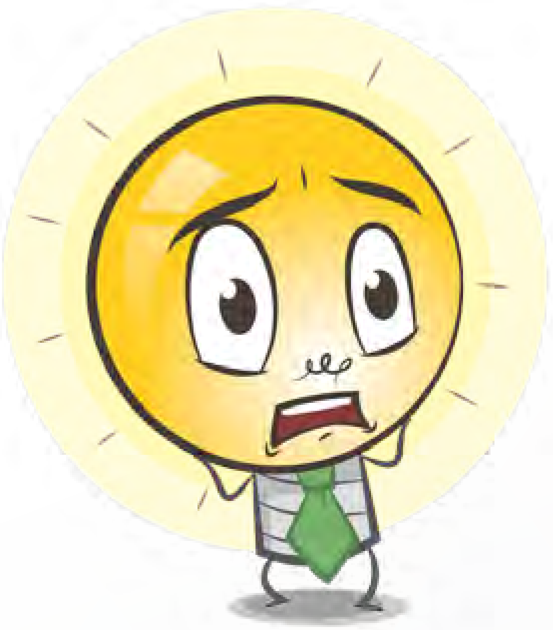

What am I doing this week?

How did my home activity go?

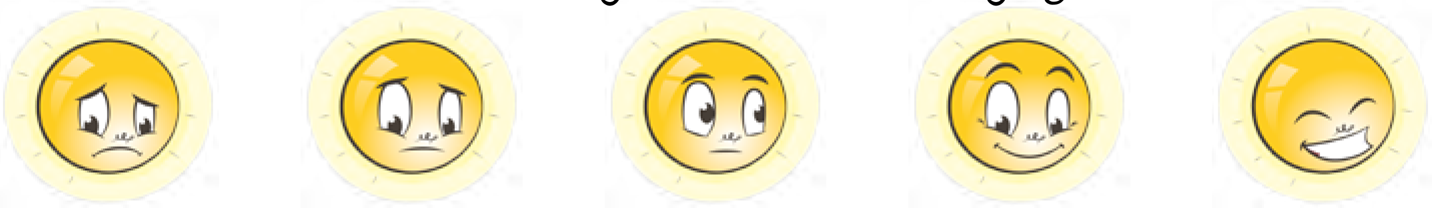

Section to be completed by parents.

Did your child complete the activity?

☐ Yes ☐ No ☐ Partially

How did your child find the activity?

☐ Very easy ☐ Easy ☐ Appropriate ☐ Difficult ☐ Very difficult

Was the goal of the activity clear to you?

☐ Yes ☐ No ☐ Partially

How did your child behave during the activity?

☐ Very restless ☐ Restless ☐ Indifferent ☐ Focused ☐ Very focused

Comments:

Date : __ / __ / __

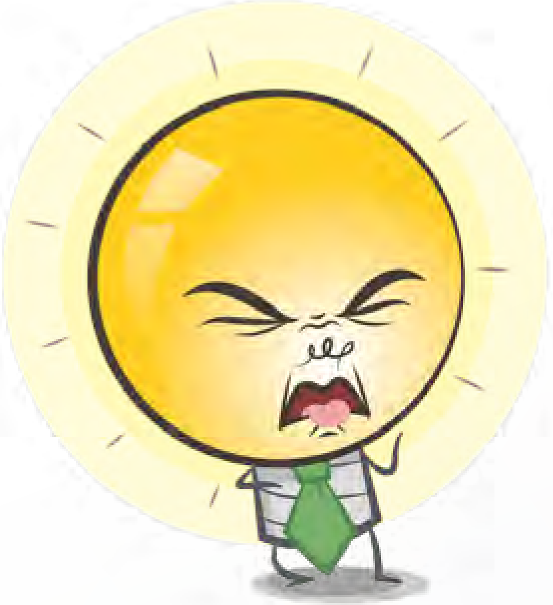

What am I doing this week?

How did my home activity go?

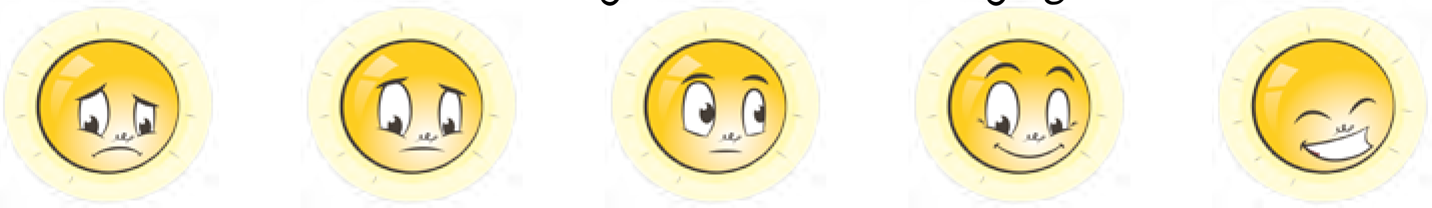

Section to be completed by parents.

Did your child complete the activity?

☐ Yes ☐ No ☐ Partially

How did your child find the activity?

☐ Very easy ☐ Easy ☐ Appropriate ☐ Difficult ☐ Very difficult

Was the goal of the activity clear to you?

☐ Yes ☐ No ☐ Partially

How did your child behave during the activity?

☐ Very restless ☐ Restless ☐ Indifferent ☐ Focused ☐ Very focused

Comments:

Date : __ / __ / __

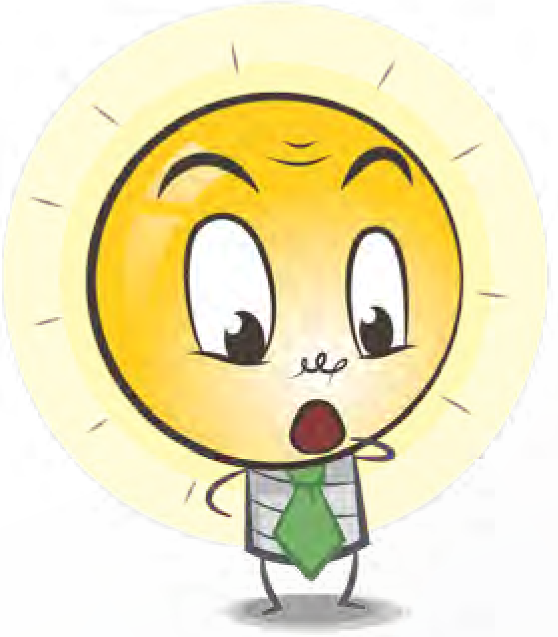

What am I doing this week?

How did my home activity go?

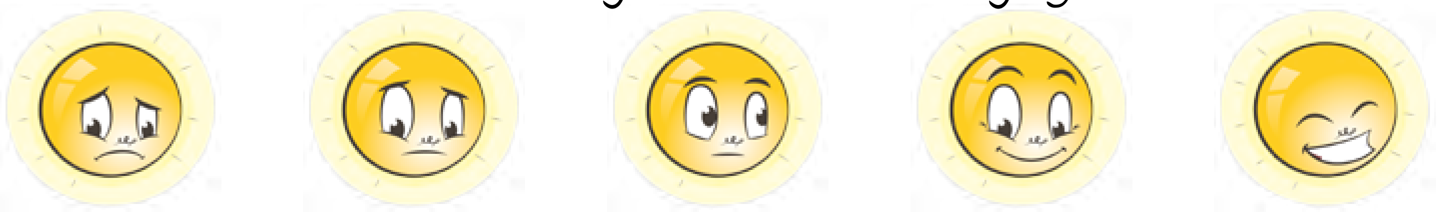

Section to be completed by parents.

Did your child complete the activity?

☐ Yes ☐ No ☐ Partially

How did your child find the activity?

☐ Very easy ☐ Easy ☐ Appropriate ☐ Difficult ☐ Very difficult

Was the goal of the activity clear to you?

☐ Yes ☐ No ☐ Partially

How did your child behave during the activity?

☐ Very restless ☐ Restless ☐ Indifferent ☐ Focused ☐ Very focused

Comments:

Date : __ / __ / __

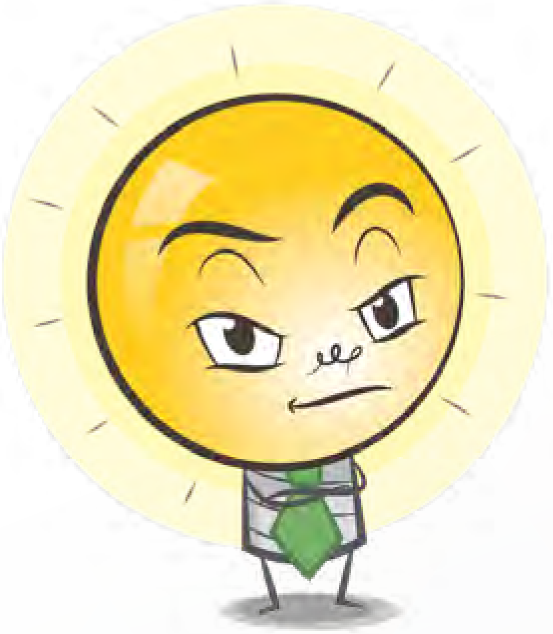

What am I doing this week?

How did my home activity go?

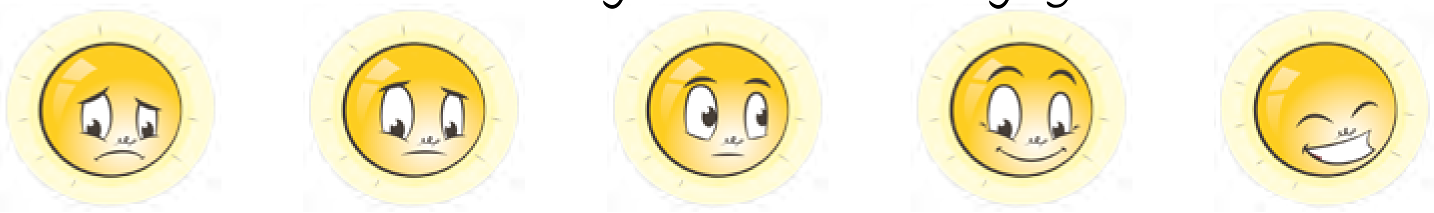

Section to be completed by parents.

Did your child complete the activity?

☐ Yes ☐ No ☐ Partially

How did your child find the activity?

☐ Very easy ☐ Easy ☐ Appropriate ☐ Difficult ☐ Very difficult

Was the goal of the activity clear to you?

☐ Yes ☐ No ☐ Partially

How did your child behave during the activity?

☐ Very restless ☐ Restless ☐ Indifferent ☐ Focused ☐ Very focused

Comments:

Date : ___ / ___ / ___

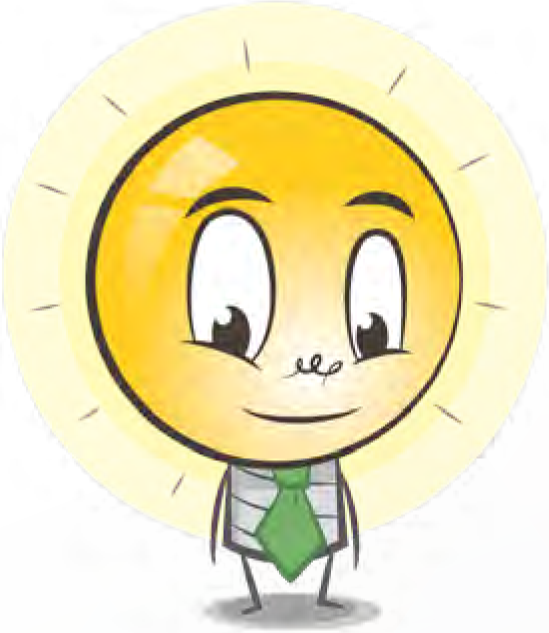

What am I doing this week?

How did my home activity go?

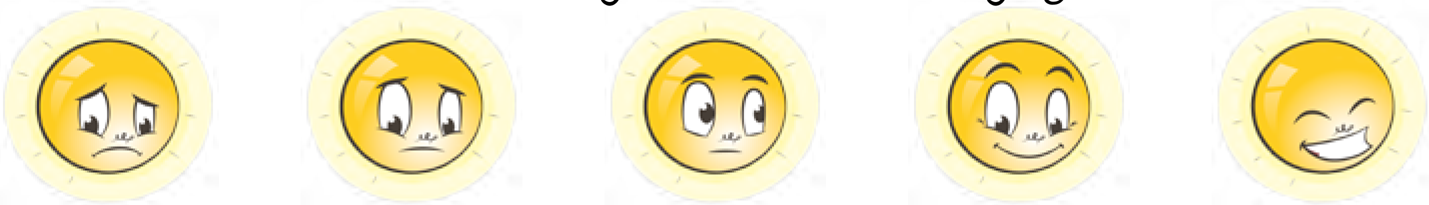

Section to be completed by parents.

Did your child complete the activity?

☐ Yes ☐ No ☐ Partially

How did your child find the activity?

☐ Very easy ☐ Easy ☐ Appropriate ☐ Difficult ☐ Very difficult

Was the goal of the activity clear to you?

☐ Yes ☐ No ☐ Partially

How did your child behave during the activity?

☐ Very restless ☐ Restless ☐ Indifferent ☐ Focused ☐ Very focused

Comments:

Date : ___ / ___ / ___

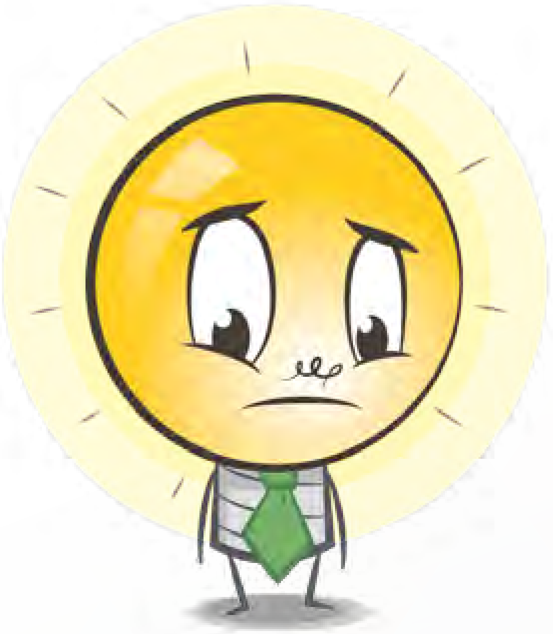

What am I doing this week?

How did my home activity go?

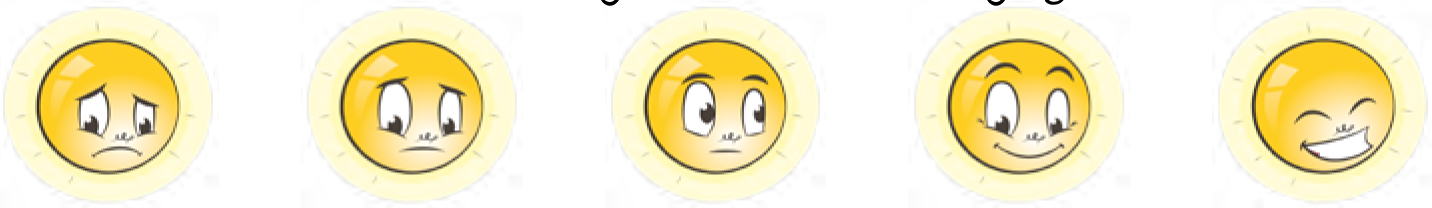

Section to be completed by parents.

Did your child complete the activity?

☐ Yes ☐ No ☐ Partially

How did your child find the activity?

☐ Very easy ☐ Easy ☐ Appropriate ☐ Difficult ☐ Very difficult

Was the goal of the activity clear to you?

☐ Yes ☐ No ☐ Partially

How did your child behave during the activity?

☐ Very restless ☐ Restless ☐ Indifferent ☐ Focused ☐ Very focused

Comments:

Date : ___ / ___ / ___

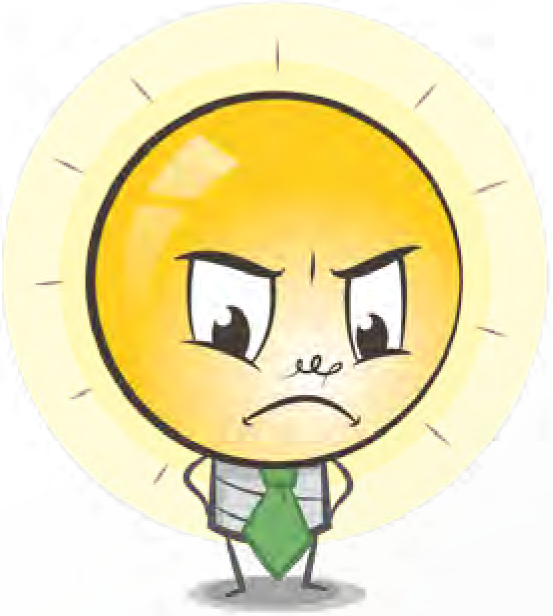

What am I doing this week?

How did my home activity go?

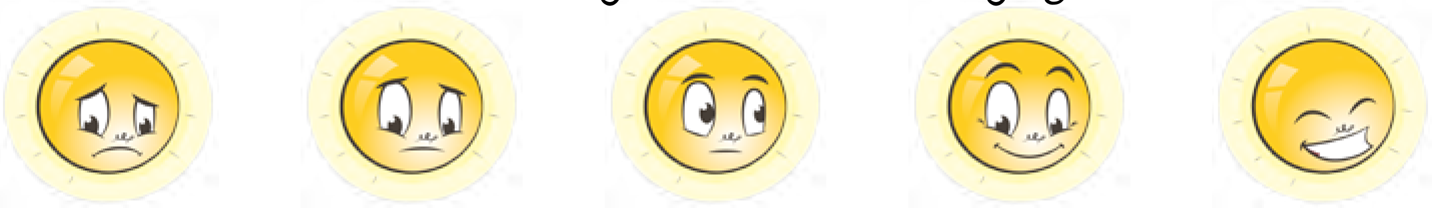

Section to be completed by parents.

Did your child complete the activity?

☐ Yes ☐ No ☐ Partially

How did your child find the activity?

☐ Very easy ☐ Easy ☐ Appropriate ☐ Difficult ☐ Very difficult

Was the goal of the activity clear to you?

☐ Yes ☐ No ☐ Partially

How did your child behave during the activity?

☐ Very restless ☐ Restless ☐ Indifferent ☐ Focused ☐ Very focused

Comments:

Date : ___ / ___ / ___

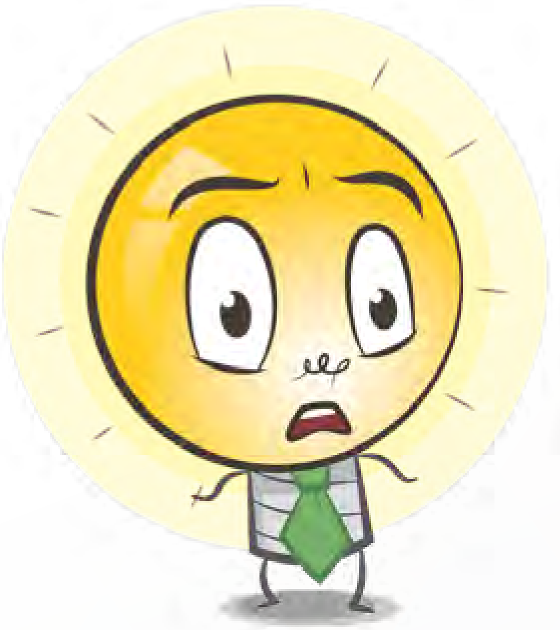

What am I doing this week?

How did my home activity go?

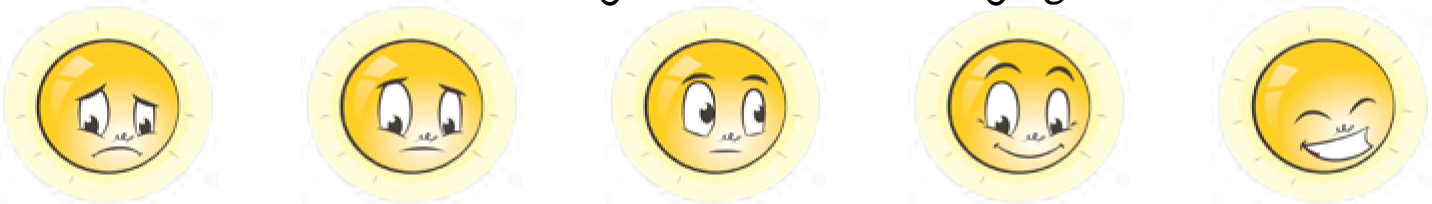

Section to be completed by parents.

Did your child complete the activity?

☐ Yes ☐ No ☐ Partially

How did your child find the activity?

☐ Very easy ☐ Easy ☐ Appropriate ☐ Difficult ☐ Very difficult

Was the goal of the activity clear to you?

☐ Yes ☐ No ☐ Partially

How did your child behave during the activity?

☐ Very restless ☐ Restless ☐ Indifferent ☐ Focused ☐ Very focused

Comments:

Date : __ / __ / __

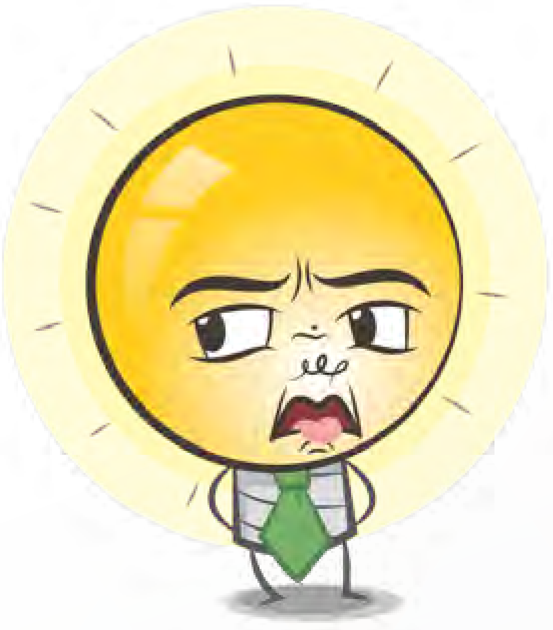

What am I doing this week?

How did my home activity go?

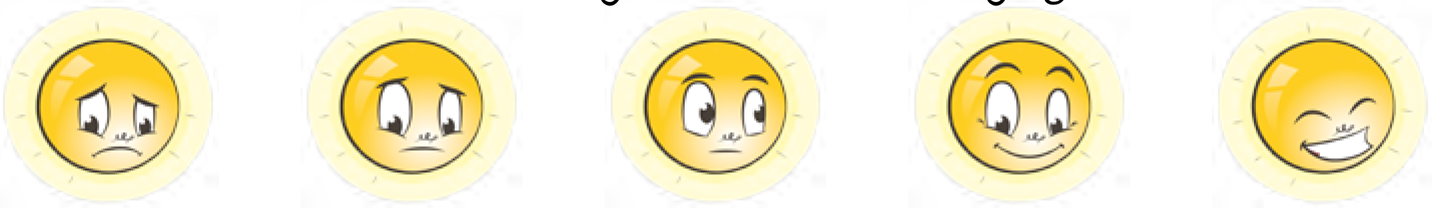

Section to be completed by parents.

Did your child complete the activity?

☐ Yes ☐ No ☐ Partially

How did your child find the activity?

☐ Very easy ☐ Easy ☐ Appropriate ☐ Difficult ☐ Very difficult

Was the goal of the activity clear to you?

☐ Yes ☐ No ☐ Partially

How did your child behave during the activity?

☐ Very restless ☐ Restless ☐ Indifferent ☐ Focused ☐ Very focused

Comments:

Date : ___ / ___ / ___

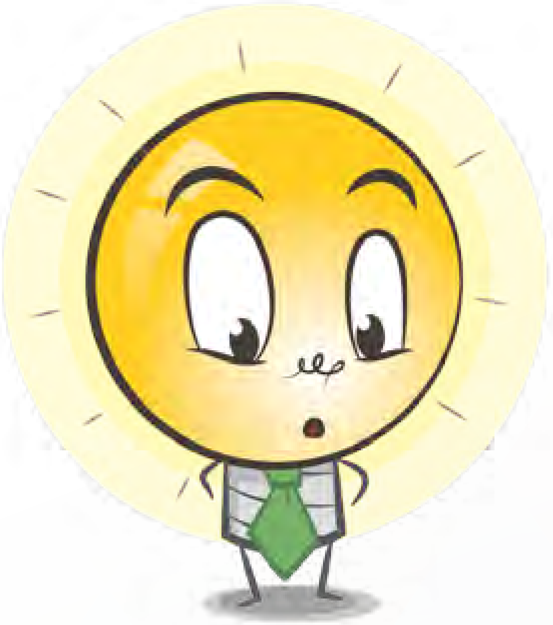

What am I doing this week?

How did my home activity go?

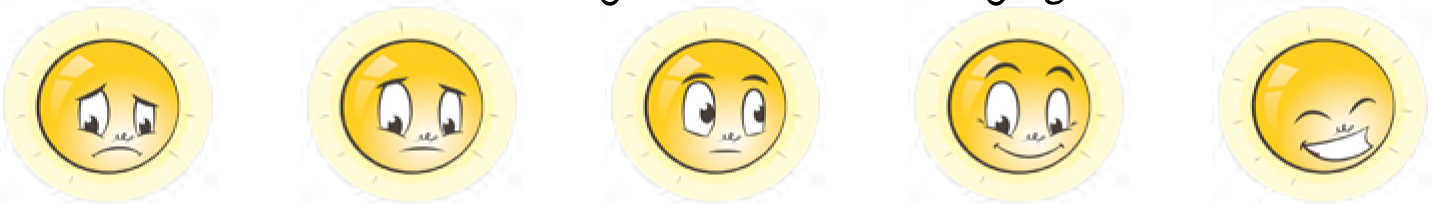

Section to be completed by parents.

Did your child complete the activity?

☐ Yes ☐ No ☐ Partially

How did your child find the activity?

☐ Very easy ☐ Easy ☐ Appropriate ☐ Difficult ☐ Very difficult

Was the goal of the activity clear to you?

☐ Yes ☐ No ☐ Partially

How did your child behave during the activity?

☐ Very restless ☐ Restless ☐ Indifferent ☐ Focused ☐ Very focused

Comments:

Date : ___ / ___ / ___

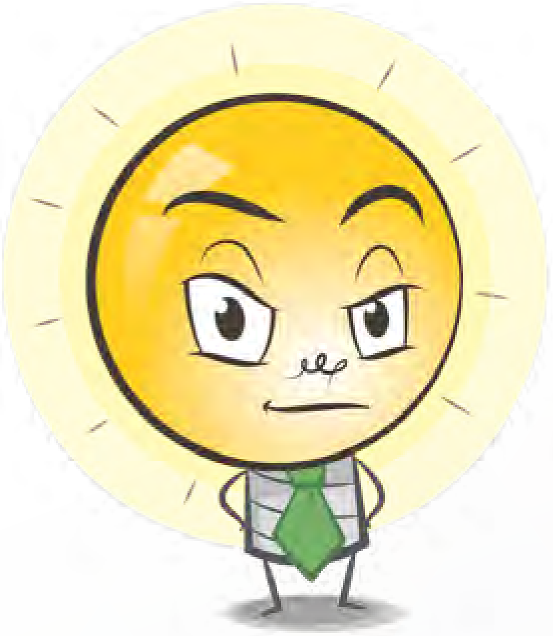

What am I doing this week?

How did my home activity go?

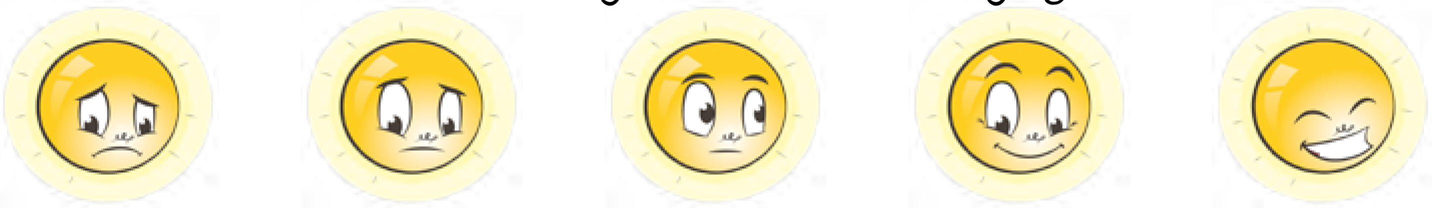

Section to be completed by parents.

Did your child complete the activity?

☐ Yes ☐ No ☐ Partially

How did your child find the activity?

☐ Very easy ☐ Easy ☐ Appropriate ☐ Difficult ☐ Very difficult

Was the goal of the activity clear to you?

☐ Yes ☐ No ☐ Partially

How did your child behave during the activity?

☐ Very restless ☐ Restless ☐ Indifferent ☐ Focused ☐ Very focused

Comments:
