# Supplementary material for: «Cognitus & Moi»: A Computer-Based Cognitive Remediation Program for Children with Intellectual Disability
Source: Front Psychiatry. 2016 Feb 3;7:10. doi: 10.3389/fpsyt.2016.00010 (PMC4737901; doi:10.3389/fpsyt.2016.00010)
Supplement: Supplementary file 2 [file image_2.pdf]

Parent Handbook

Cognitive functions

Cognitive functions allow us to understand our environment and adapt our behavior according to the situations we encounter. The five main cognitive functions are: attention, memory, executive functions (organization, flexibility, reasoning), visuospatial abilities, and social cognition.

We use these functions to acquire academic and professional knowledge and gain independence.

Your child suffers from cognitive impairments affecting his or her daily life, diagnosed through a neuropsychological assessment.

Cognitive remediation

Cognitive remediation aims to alleviate your child's cognitive difficulties in order to help him or her to better cope with the demands of daily life.

Cognitive remediation uses your child's strengths in order to develop strategies to compensate for his or her weaknesses.

Cognitus & Me

This program is primarily focused on attention and visuospatial functions.

It is divided into 16 one-on-one sessions with us. Your child will complete both computer and pencil-and-paper exercises.

To facilitate the transfer of these strategies into daily life, your child will have activities to perform at home between sessions. Your child should ask for your help in completing these activities.

There are no right or wrong strategies for performing activities at home. Positive reinforcement is a key factor in cognitive remediation. It is provided by the therapist, Cognitus, and by you, the parent.

The home activity book is used to make the connection between work completed during sessions with activities to be performed at home. This is an individual workbook, unique to your child. Your child may personalize it as he or she desires.

Attention

Attention allows us to focus on the information needed to perform an activity (the shape of an object, the smell of a meal, a teacher's instructions). It also allows us to avoid becoming distracted by surrounding information (I can focus on reading without becoming distracted by my brother who is making noise playing cars next to me).

A child with an attention deficit cannot stay focused on the same activity for long periods of time, has a hard time finishing time-consuming activities, often misplaces his or her belongings, struggles to stay still, and is easily distracted.

Visuospatial functions

Space is defined by our surroundings: the paper on which I'm writing, the classroom, the person next to me, etc. and by own position (my right, my left, in front, behind).

Visuospatial skills are a set of processes that allow us to analyze and understand visuospatial information in our environment. They are necessary to geometry, geography, taking public transport, driving, etc.

A child with visuospatial difficulties has a hard time navigating, gets lost in the school hallways, struggles to locate important information on a worksheet, has trouble writing between the lines, etc. These difficulties may be a source of anxiety.
