# Supplementary material for: «Cognitus & Moi»: A Computer-Based Cognitive Remediation Program for Children with Intellectual Disability
Source: Front Psychiatry. 2016 Feb 3;7:10. doi: 10.3389/fpsyt.2016.00010 (PMC4737901; doi:10.3389/fpsyt.2016.00010)
Supplement: Supplementary file 3 [file image_3.pdf]

# Certificate

First prize in cognitive remediation
awarded to:

________________________

By Cognitus

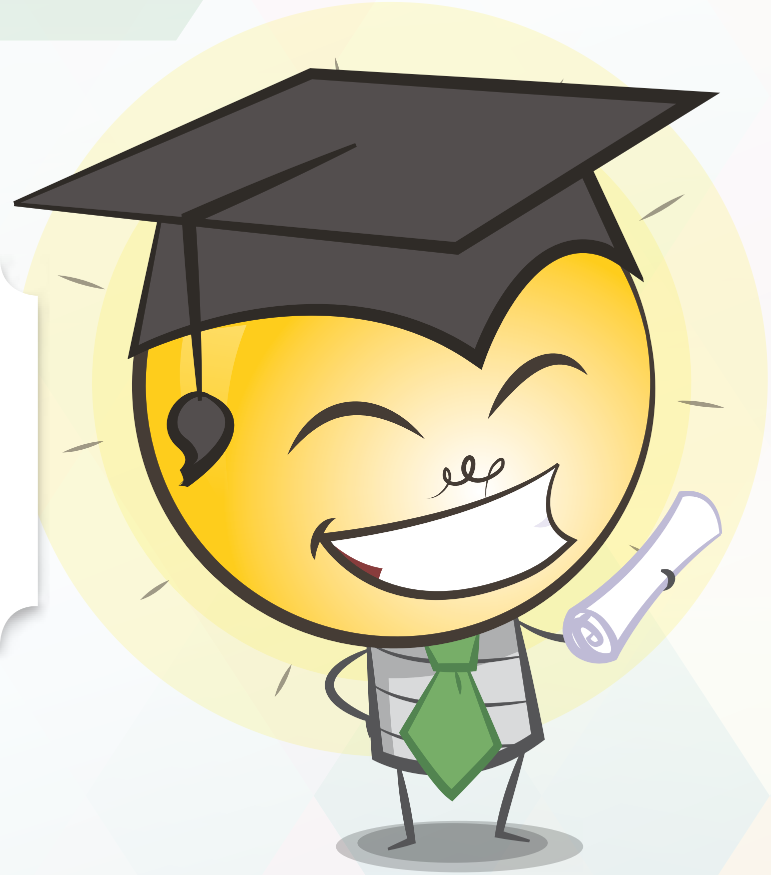

Graduate's signature

Cognitus' signature

Cognitus
