# Supplementary figures and images for: «Cognitus & Moi»: A Computer-Based Cognitive Remediation Program for Children with Intellectual Disability
Source: Front Psychiatry. 2016 Feb 3;7:10. doi: 10.3389/fpsyt.2016.00010 (PMC4737901; doi:10.3389/fpsyt.2016.00010)

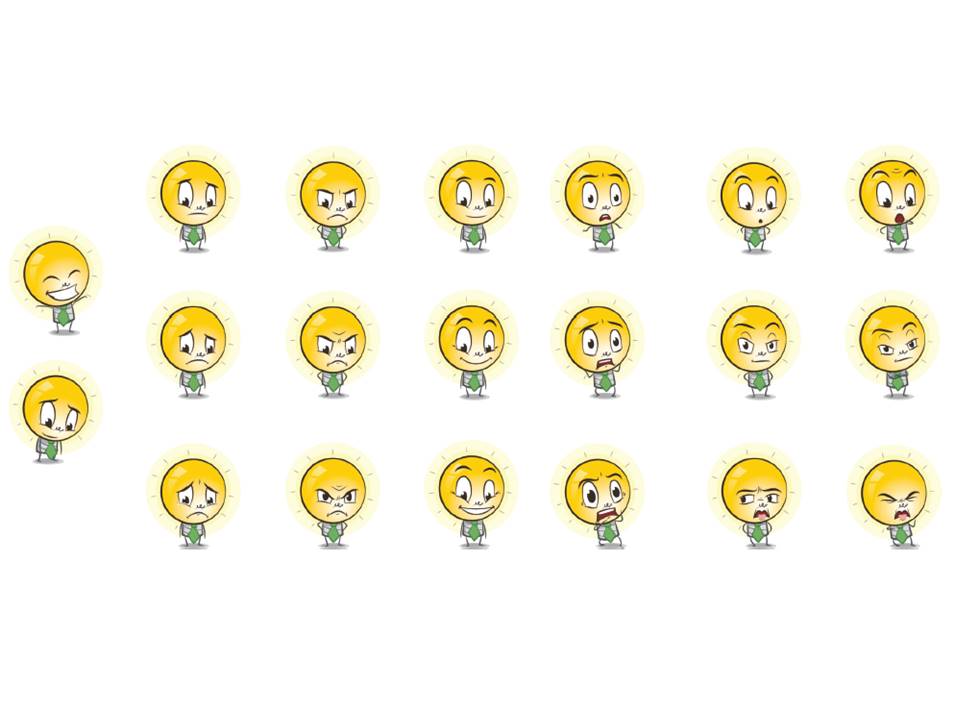

Supplement: Supplementary file 4 [file image_4.jpeg]
